# A Review of Functional Motifs Utilized by Viruses

**DOI:** 10.3390/proteomes4010003

**Published:** 2016-01-21

**Authors:** Haitham Sobhy

**Affiliations:** Department of Molecular Biology, Umeå University, 901 87 Umeå, Sweden; haithamsobhy@gmail.com or haitham.sobhy@umu.se; Tel.: +46-90-785-67-81

**Keywords:** clathrin endocytosis, low-complexity repeats, ubiquitylation, agnoprotein, APOBEC, pentraxin, PDZ domain, retinoblastoma, inhibitor of apoptosis (IAP), transposition

## Abstract

Short linear motifs (SLiM) are short peptides that facilitate protein function and protein-protein interactions. Viruses utilize these motifs to enter into the host, interact with cellular proteins, or egress from host cells. Studying functional motifs may help to predict protein characteristics, interactions, or the putative cellular role of a protein. In virology, it may reveal aspects of the virus tropism and help find antiviral therapeutics. This review highlights the recent understanding of functional motifs utilized by viruses. Special attention was paid to the function of proteins harboring these motifs, and viruses encoding these proteins. The review highlights motifs involved in (i) immune response and post-translational modifications (e.g., ubiquitylation, SUMOylation or ISGylation); (ii) virus-host cell interactions, including virus attachment, entry, fusion, egress and nuclear trafficking; (iii) virulence and antiviral activities; (iv) virion structure; and (v) low-complexity regions (LCRs) or motifs enriched with residues (Xaa-rich motifs).

## 1. Introduction

Interactions between viral and cellular proteins are required for virus entry, replication, or egress from the cell. These interactions are facilitated by peptide sequences, so-called domains or motifs [1,2]. These sequences could be either (i) short linear motifs (SLiM), 3–11 residues, e.g., RGD; (ii) structural motifs or domains, about 30 residues, e.g., tetratricopeptide repeat (TPR), zinc finger or ankyrin; or (iii) they may contain a repeated residue(s) (e.g., Leu-rich, SR-rich, AR-rich or PEST-rich motifs). The consensus motif follows the PROSITE pattern [3]. The consensus is formed of a regular expression pattern, e.g., Px(2)[ED]. In the pattern, a single-letter amino acid abbreviation is indicated. The alternative (degenerated) residues in a position are bracketed, while “x” letter denotes any residue in the position. The number between parentheses refers to the number of occurrences of a residue.

**Figure 1:**
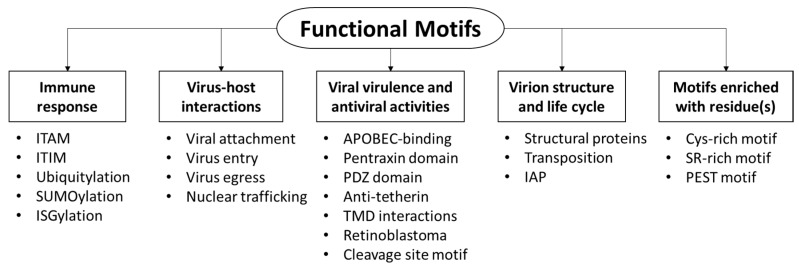
Five categories of motifs were reviewed, based on function of proteins harboring the motif.

Viruses utilize a number of functional motifs to attach and enter into host cells, or interact with cellular proteins. This article aims to review the current understanding of motifs utilized by viruses for fruitful infection, highlighting the function of motifs and/or proteins harboring these motifs, in an attempt to classify the motifs based on the molecular function of the harboring proteins. The motifs can be classified into five main categories (Figure 1): (i) motifs that mediate immune response; (ii) virus-host interactions, including entry and cellular trafficking; (iii) virulence and antiviral activities, which may disturb cellular processes; (iv) virion structure; and (v) motifs enriched with residues.

## 2. Motif Involved in Immune Response and post-translational modification processes

**Immune response.** B and T cells employ two types of receptors with positive and negative regulators, the so-called immunoreceptor tyrosine-based activation motif (ITAM) and the immunoreceptor tyrosine-based inhibition motif (ITIM), respectively [4]. These receptors are responsible for immune response and signal transduction in immune cells. They bear either ITAM (Yxx[LI]x_6–8_Yxx[LI]) or ITIM ([SIVL]xYxx[IVL]) motifs. The dendritic cell (DC) immunoreceptor (DCIR), a C-type lectin receptor expressed on DCs, acts as an attachment factor for human immunodeficiency virus type 1 (HIV-1) [5]. DCIR contains ITIM, which binds to the Glu-Pro-Ser (EPS) motif. Chemical inhibitors directed against this motif prevent attachment of HIV-1 to DCs.

**Post-translational modification processes.** Cellular processes, such as ubiquitylation, SUMOylation and ISGylation, require particular motifs for proteins to bind and initiate them. In adenoviruses, protein VI recruits Nedd4 E3 ubiquitin ligases by the PPxY motif, facilitating its ubiquitylation [6]. The SLQxLA, VxHxMY, HCCH (Hx_5_Cx_17–18_Cx_3–5_H) and PPLP motifs in the viral infectivity factor (Vif) protein bind to Cullin5, ElonginB and C, inducing protein polyubiquitination and proteasome-mediated degradation [7,8,9,10].

**SUMOylation** is a post-translational modification process by which small protein (SUMO, small ubiquitin-related modifier) binds to a wide range of cellular proteins, modifying their functions by adding a bulky moiety, and promoting particular protein-protein interactions [11,12]. SUMOylation of substrates is initiated by the binding of SUMO with lysine residue in the SUMOylation consensus motif, φKx[DE], where φ denotes large hydrophobic residues (F, I, L or V). It is noteworthy that the SUMO motif is not the exclusive motif for SUMOylation, and the SUMO substrate can be modified in different sites, such as the SxS (φφxSxS[DE][DE][DE]) and [VI]x[VI][VI] motifs [12,13,14]. A number of viruses (including herpesviruses and hepatitis C virus, HCV) were able to trigger SUMOylation-dependent mechanisms by recruiting E2 and E3 ubiquitin ligases [15,16,17,18]. SUMO was suggested to play roles in the nuclear localization of viral cargo [19], suggesting their roles in virus replication [17]. Notably, the sentrin-specific proteases (SENPs) family are SUMO proteases, which are able to detach SUMOs from their substrates [20]. Interfering with the proteins involved in (de-)SUMOylation processes via SENPs was suggested as a potential technique for developing an antiviral agent [17,18,21].

Viral proteins, such as paramyxovirus C and V proteins, mouse cytomegalovirus (CMV) pM27, and Kaposi's sarcoma-associated herpesvirus K3, K5 and viral interferon regulatory factor 3, can inhibit signal transduction and activators of transcription (STAT) or major histocompatibility complex [22,23,24,25,26,27,28,29,30]. These interactions downregulate the interferon (IFN) pathway, regulate the expression of interferon-stimulated genes (ISGs), and suppress both cytokine-mediated immunity and anti-viral defense [22]. Similar mechanisms were suggested for equine herpesvirus-1 [31], hepatitis E virus [32], and hepatitis B virus [33].

ISG15, a ubiquitin-like interferon-stimulated protein, is stimulated by interferon or viral infection [34,35]. ISG15 is cytokine-like protein that promotes antiviral immune response. On mice, ISG15 expression reduces Sindbis virus replication and clearance in multiple organs, and attenuates infection [34]. Further evidence shows that *Novirhabdovirus*, *Birnavirus* and *Iridovirus* infection could be inhibited by the over-expression of zebrafish ISG15 in EPC cells [36,37]. On the other hand, ISG15 conjugates with the substrate protein through its conserved LRLRGG consensus sequence, leading to antiviral response [35]. Mutations of glycine residues (LRAA) destabilize this conjugation [36]. However, evidence shows that the fish ISG15 homolog can promote an antiviral immune response, even in unconjugated form [37].

## 3. Motifs Required for Virus Attachment, Entry, Trafficking, and Egress

### 3.1. Viral Receptors

Viruses utilize receptors and co-receptors to attach and enter into host cells. HIV attaches to one or two co-receptors, CCR5 or CXCR4, to enter cells [38,39,40,41,42,43,44,45]. The conserved GPG[RQ] motif in the crown of the third variable loop region of the gp120 protein is crucial for virus attachment [43,44,45,46,47]. In adenovirus (Adv), it is suggested that the KKTK motif in Adv2 and Adv5 fiber shaft attaches to heparin sulfate proteoglycans to start the infection [48,49]. A mutation in KKTK affects Adv5 tropism. Further investigations show that the KKTK motif in Adv-C is important for post-entry steps [50,51]. Virus lacking the KKTK motif efficiently infects liver cells *in vivo*.

**Integrin-binding**. Integrins are cell surface adhesion molecules composed of α and β subunits. They are expressed by a variety of cells and can be utilized by microbes [49,52]. Integrins interact with the conserved Arg-Gly-Asp (RGD) motif of the adenovirus penton base, which promote endocytosis and endosomal escape, as reviewed in [53,54]. Several reports suggest the ability of viruses to evolve mechanisms by which they utilize RGD-like motifs (RGG or GGG), as reviewed in [55] or the potential integrin-binding motif YGD motif [56] to enter into host cells. Moreover, the SDI motif in glycoprotein H (gH) of equine herpes viruses 1 and 4 may bind to integrins [57]. Foot-and-mouth disease virus (FMDV) VP1 capsid protein harbors the RGDLxxL sequence, which is required for binding to cellular integrins [58]. The two Leu residues stabilize the interaction and play roles in determining integrin specificity. Nonetheless, in the absence of RGD, DLxxL, KGD or KGE is employed for the attachment to cellular receptors [58].

### 3.2. Virus Entry

#### 3.2.1. Endocytosis

The 3a protein encoded by severe acute respiratory syndrome–associated coronavirus (SARS-CoV) functions as an ion channel protein [59]. It harbors the Yxxφ motif, which is necessary for endocytosis, intracellular trafficking, and surface transport of SARS-CoV. Sodium taurocholate co-transporting polypeptide (NTCP) at the plasma membrane is a receptor for hepatitis B and D viruses (HBV and HDV) [60]. Endocytosis of HBV and HDV is regulated by the dileucine motif (^222^LL^223^) and the phosphorylation of T^225^ and S^226^ in NTCP [61]. Moreover, PPxY is required for Adv5 entry and cellular microtubule-dependent trafficking [6].

#### 3.2.2. Clathrin Endocytosis

The clathrin-coated vesicles recruit soluble clathrin by adaptor proteins (APs) AP-1 (in the *trans*-Golgi network) and AP-2 (at the cell surface). The clathrin-binding motifs of APs bind to the N-terminal domain of clathrin. Two clathrin-binding motifs were defined: clathrin-box, which conforms to sequence LφXφ[DE] or L[LI][DEN][LF][DE], and W-box, which conforms to sequence PWxxW [62]. Moreover, the µ subunit of AP1 recognizes two sorting signals, a tyrosine-based Yxxφ motif and an acidic dileucine motif, [ED]xxxL[LI] [63]. HIV-1 viral protein unique (Vpu) hijacks AP-1 and antagonizes BST2 via YxYxxφ, [63]. AP-1 reroutes BST2 to the lysozyme and mediates the endo-lysosomal degradation of BST2. Similar mechanisms were described in HIV Nef, which hijacks clathrin AP-1 and interacts with the major histocompatibility complex (MHC-1) [64,65]. This interaction is stabilized by (PxxP)_3_ repeats and directs MHC-I to the endo-lysosomal pathway.

#### 3.2.3. Virus Fusion

The short motif mediates interaction with other proteins leading to virus fusion and entry. For example, the fusion protein encoded by the Newcastle disease virus (NDV) harbors LL and Yxxφ motifs in the cytoplasmic tail and plays a role in viral fusion, replication and pathogenesis [66,67]. Moreover, interferon-induced transmembrane (IFITM) proteins inhibit virus entry and cell-cell fusion of several viruses, including coronavirus, HIV-1, influenza and Ebola viruses [68]. The KRxx (dibasic residues) motif in the C-terminal of IFITM-1 modulates a species-specific antiviral sorting signal against viruses by controlling protein subcellular localization, while IFITM-3 interacts with AP2 through its Yxxφ sorting motif at the N-terminus [69,70,71].

### 3.3. Virus Egress from the Cell

**Figure 2:**
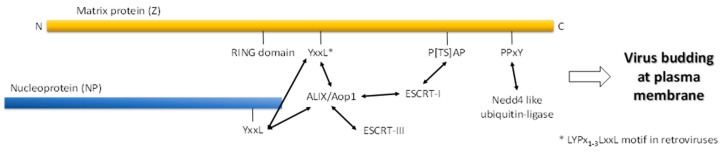
A schematic diagram of arenavirus late-domain motifs and their role in interaction with cellular proteins leading to virus budding and egress from the cell [67].

Viruses recruit endosomal sorting complexes required for the transport (ESCRT) pathway to egress from the cell, which leads to virus budding and initiating new infection, as reviewed in [72,73,74,75,76]. The pathway is mediated by several molecular interactions between proteins through late (l)-domain motifs (P[TS]AP, PPxY, YxxL, and φPxV) (Figure 2) [67,77,78]. These motifs mediate binding to ESCRT, which leads to the budding and release of viruses, including a number of retroviruses, arenaviruses and paramyxoviruses. In the absence of the PPPY motif, LYPx_n_L in the gag protein serves as an alternative motif that recruits ESCRT machinery for the release and replication of retroviruses [79,80], while in Ebola virus, these interactions are mediated by ^7^PTAP^10^, ^10^PPEY^13^ and ^18^YPx_n_[LI]^26^ [81]. First, proteins harboring the PPxY, LYPx_n_L or PTAP motifs interact with Nedd4, Alix and Tsg101 proteins, respectively. Then, these interactions trigger ESCRT machinery and the release of the virus by budding [82]. Interestingly, archaeal ESCRT could be involved in the egress of *Sulfolobus* turreted icosahedral virus by forming virus-associated pyramid structures on the cell membrane of *Sulfolobus* Archaea, as reviewed in [83]. Due to the crucial role of these motifs, several attempts were suggested for developing antiviral therapeutic agents targeting these motifs and/or the proteins harboring them [78,81]. Targeting l-domain-dependent recruitment of host Nedd4 and Tsg101 shows depletion of viral egress for a number of RNA viruses, including vesicular stomatitis, rabies viruses, and hepatitis E virus [84,85].

### 3.4. Nuclear Trafficking

The trafficking of a protein into or from the nucleus is orchestrated by two motifs: (i) nuclear export signal (NES), which regulates proteins export from the nucleus to the cytoplasm; and (ii) the nuclear localization sequence (NLS) motif, which imports proteins into the nucleus [86,87]. The canonical NES consensus motif is LxxxLxxLxL, but L can be replaced by I, V, F or M [88], whereas the NLS motifs are classified into six classes (as seen below in Table 1 and Table S1) [89]. Interestingly, the first NLS was discovered in SV40 Large T-antigen with the monopartite PKKKRKV sequence [90,91,92]. The nucleoprotein of influenza B virus (BNP) harbors a conserved ^44^KRxR^47^ motif, and a mutation on the K or R residue results in the disruption or failure of nuclear import and localization, suggesting that the motif is a NLS sequence [93,94]. 

**Table 1:** List of pattern of functional motifs and the function of the protein harboring them. ^1^

Function of Protein Containing the Pattern	Pattern Motif	References
6-cysteine motif, degradation of chitin and chitotriose	Cx_13–20_Cx_5–6_Cx_9–19_Cx_10–14_Cx_4–14_C	[95,96]
Adenovirus fiber flexibility motif	KLGxGLxF[DN] and KxGGLxF[DN]	[50]
Agnoprotein function, productive viral infection	L[FL][VI]F[VIL]LE[LF]LLxF and Qxx[IML]xx[FY]	[97,98,99]
Agnoprotein—NLS	RRRRx_5_Rx_4_RK	[100]
Binding of virus proteins to retinoblastoma protein, gene expression and virus replication	LxCxE and [LI]xCx[DE]	[101,102,103,104,105,106,107,108,109]
Binding to ESCRT, paramyxoviruses budding	φPxV	[79]
Binding to integrins and viral attachment to cellular receptors	RGD, DLxxL, LDV, RGDLxxL, SDI, KGD and KGE	[53,54,55,56,57,58]
Budded virions production and nucleocapsid assembly	Cx_5_Cx_n_Hx_6_C (C2HC zinc finger)	[95,96]
Clathrin-binding motifs, clathrin-box	LφXφ[DE], L[LI][DEN][LF][DE] and PWxxW	[62]
Cleavage motif of Newcastle disease virus	[GE][KR]Q[GE]RL and [RK]RQ[RK]RF	[110]
Cleavage site for Influenza A virus hemagglutinin	KKKRGLF, [QE][ST]RGLF, Rx[RK]RGLF, RxRRGLF and RxxRGLF	[111]
Enhance virion-release, anti-tetherin activity	DSGxxS	[112,113]
Helix-Helix Interactions	AxxxAxxxAxxxW and VxxxIxxLxxxL	[114,115]
Heparan sulfate-binding motif, post-internalization steps of adenovirus	KKTK, or bbxb and bbbxxb	[48,49,50,51]
HIV neutralization by human antibodies	GPG[RQ]	[43,44,45,46,47]
HIV release, interfering with tetherin function	[GD]DIWK	[113]
Induction of cellular-malignant transformation by Kaposin, activation of cap-dependent translation, and HIV retrotransposition	LxxLL	[116,117,118,119,120,121,122]
IAP, block the apoptosis	Gx_2_Yx_4_Dx_3_Cx_2_Cx_6_Wx_9_Hx_6–10_C, Cx_2_Cx_9–39_Cx_1–3_Hx_2–3_Cx_2_Cx_4–48_Cx_2_C and A[KITV][AEP][FEISY]	[123,124,125]
Interact with clathrin adaptor protein	PxxP and YxYxxΦ	[63,64,65]
ISGylation, antiviral response	LRGG and LRLRGG	[35,36]
ITAM motif	Yxx[LI]x_6–8_Yxx[LI]	[4]
ITIM motif	[SIVL]xYxx[IVL]	[4]
Necessary for endocytosis, intracellular trafficking, interact with clathrin APs, and promotes viral spread, fusion and replication	YxxΦ	[64,65]
Nuclear export signal (NES), regulates protein export to nucleus from cytoplasm	[LIVFM]x_2–3_[LIVFM]x[LIVFM] and LxxxLxxLxL	[88]
NLS motifs	i: KR[KR]R and K[KR]RK ii: [PR]xxKR{DE}[KR] iii: KRx[WFY]xxAF iv: [RP]xxKR[KR]{DE} v: LGKR[KR][WFY] Bipartite: KRx_10–12_K[KR][KR] and KRx_10–12_K[KR]X[KR]	[89,93,94]
Pentraxin domain, pathogen recognition, host defense, and antiviral response	HxCx[ST]WxS	[126,127]
Protein folding, Rossmann folds motifs, and bind FAD or NAD(P)	Gx_3_G, Gx_3_[GA] and Gx_1-2_GxxG	[128]
Protein interaction and thiol-disulfide transfer	CxxC and CxxxC	[129,130,131]
Proton transport, channel function, and transmembrane domain	HxxxW	[132]
Recruits ESCRT pathway, and mediates viral budding and release	YxxL, P[TS]AP and LYPxL	[67,77,79]
Regulation by interaction of retrovirus Vif with APOBEC, cullin5, elongin, and E3 ligase	PPLP, SLQxLA, VxHxMY, HCCH, YYxW, DPD, YxxL, YRHHY, EDRW, DRMR, TGERxW, LGxGxxIxW, WxSLVK, W[HKN]SLVK, VxIPLx_4-5_L, VxIPLx_4-5_Lxφx_2_YwxL, SL[VI]x_4_Yx_9_Y and T[DEQ]x_5_Adx_2_[IL]	[7,8,9,10,133,134,135,136,137,138,139,140,141,142,143,144]
Sorting signal, anti-tetherin	ExxxLV	[145]
SUMOylation—SUMO binding to substrate	φφxSxS[DE][DE][DE], φKx[DE] and [VI]x[VI][VI]	[12,13]
Ubiquitylation, interaction with Nedd4 E3 ubiquitin ligases, recruit ESCRT pathway, and mediates virus entry, cellular microtubule-dependent trafficking, budding, and release	PPxY	[6,67,77]

^1^ Degenerate residues are bracketed, braces refer to the excluded residues (*i.e.*, any residues except those between braces), “x” means any residue, b refers to basic residues (H, K or R), “φ” denotes large hydrophobic residues (F, I, L or V), and the number of recurrence is indicated after residues.

#### Agnoprotein

Agnoprotein (*agnosis* means unknown in Latin) is a regulatory protein encoded by some polyomaviruses, including the BK virus (BKV, named after the isolation from patient, initials B.K.), JC virus (JCV, John Cunningham virus) and simian vacuolating virus 40 (SV40) [100]. The exact function is unknown, but it is reported to have role in viral DNA replication and transcription, which requires an FIL-rich motif (L[FL][VI]F[VIL]LE[LF]LLxF) at the N-terminus [97,98]. Moreover, it may facilitate nuclear egress by interacting with heterochromatin protein 1 at the nuclear envelope [146]. Interactions with proliferating cell nuclear antigen (PCNA) lead to the inhibition of PCNA-dependent DNA synthesis and the reduction of cell proliferation [99]. The PCNA-interacting protein box (PIP motif, Qxx[IML]xx[FY]) is shared with most of the PCNA-interacting proteins. Although JCV, BKV and SV40 agnoproteins harbor PIP-like consensus (QR[LI][FL][IV]F), several regions could be involved in the interaction [99]. The agnoproteins contain a l-rich and KR-rich motif (such as RRRRx_5_Rx_4_RK), which may represent a classic NES and NLS, respectively [100]. Ironically, although agnoproteins contain NES and NLS motifs, most of the known agnoproteins localize in the cytoplasm and/or are perinuclear [100], and their nuclear trafficking needs to be elucidated.

## 4. Viral Virulence

### 4.1. APOBEC-Binding Motifs

The “Apolipoprotein B mRNA editing enzyme, catalytic polypeptide-like” (APOBEC) proteins are crucial for the editing of cytosine to uracil bases during reverse transcription (mRNA editing), as reviewed in [8,133,147,148]. Three proteins, APOBEC-3C, 3F and 3G (A3C, A3F and A3G), exhibit potent antiviral activity by inhibiting retroviruses, including HIV replication, reverse transcription and DNA integration into the host genome [147]. Vif proteins encoded by HIV and simian immunodeficiency virus (SIV) bind to E3 ubiquitin ligase, cullin5 and elongin, leading to A3 ubiquitination and proteasomal degradation [8,9,10,133,134,148]. By this mechanism, retroviruses can suppress A3 antiretroviral activity [133]. These interactions are mediated by a number of motifs, including the YRHHY, PPLP, DRMR, and T[DEQ]x_5_Adx_2_[IL] motifs, whereas other motifs were also reported (Table 1) [133,134,135,136,137,138,139,140,141,142,143,144,149]. 

### 4.2. Pentraxin Domain

The Pentraxin superfamily are pattern recognition receptors, which include long pentraxin-3 and the short serum amyloid P component and C reactive protein. They have a diverse role in inflammation, host defense and antiviral response [126,127]. These proteins are characterized by a pentameric structure and the pentraxin domain (HxCx[ST]WxS). The hemagglutinin (HA) glycoprotein of influenza A virus recognizes sialic acid on pentraxin-3, resulting in virus neutralization [150]. Further analysis suggests that this interaction is critical for productive viral infection [151].

### 4.3. The PDZ Domain

PDZ is an abbreviation for post-synaptic density protein (PSD95), *Drosophila* disc large tumor suppressor (Dlg1), and zonula occludens-I protein (zo-1). The canonical PDZ domains harbor the conserved carboxylate-binding loop motif groove ([RK]xxx[GSTF]φGφ) between αB and βB structural elements [152]. It mediates protein-protein interaction, phosphorylation and regulates cellular signaling, including transport and ion channel signaling, as reviewed in [152]. It also mediates interactions between cytoplasmic proteins and tight junction proteins, which can be used by viruses to enter into host cells, as reviewed in [153,154]. PDZ domains are classified into three classes based on the C-terminus recognition sequence motif of their target proteins: the class I domain, which recognizes the [ST]xφ motif; the class II domain, which recognizes the φxφ motif; and the class III domain, which recognizes the [DE]xφ motif.

**Figure 3:**
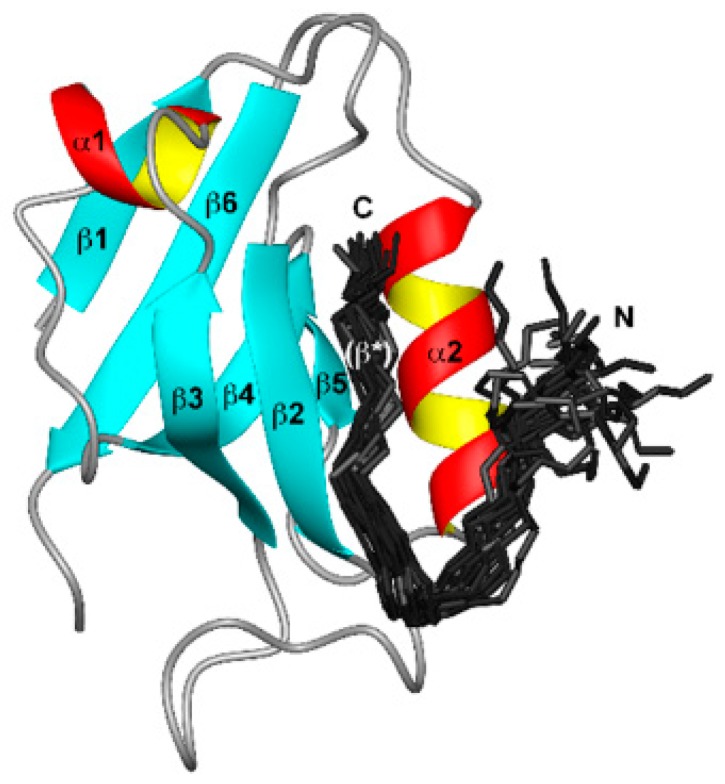
Binding of HPV E6 to the second PDZ domain (PDZ2) from the human homologue of the *Drosophila* discs large tumor suppressor protein (hDlg). E6 (150 residues) consists of two zinc-binding domains (Cx_2_Cx_29_Cx_2_C). The bundle of 20 best E6 structures (residues 141 to 151, dark grey). Adopted and modified from [156], published under Creative Commons Attribution license.

The human papillomavirus (HPV) E6 protein targets PDZ domain–containing proteins, which are regulated by protein phosphorylation and protein kinase signaling pathways, as shown in Figure 3 [155,156]. Influenza A virus NS1 contains PDZ domain–binding motif (ESEV and RSKV motifs in the NS1 of avian and human influenza viruses, respectively). A mutation in ESEV affects the PI3K/Akt pathway, interactions of NS1 with scaffolding proteins and the virulence of avian H5N1 influenza viruses [157]. Tax1 is another PDZ-binding motif containing oncoprotein, encoded by Human T-cell leukemia virus (HTLV-1) [158]. The Tax1 protein is involved in various functions, including interaction with proteins (it harbors PDZ) involved in cell signaling, such as transcription factors (cAMP response element-binding protein), nuclear factors (NF-κB), chromatin-modifying enzymes, GTPases and kinases (MAPK). These signal cascades may lead to the inhibition of cell cycle progression, and DNA repair, as reviewed in [158] and [159]. Tax1 acts as a transcriptional activator by activating PI3K-Akt and NF-κB pathways, which induce transformation, continued cell cycle progression and resisting apoptosis [159,160], and may induce CD83 expression on T cells [161].

### 4.4. Anti-Tetherin Activity

Tetherin (bone marrow stromal antigen 2, BST2) is a cellular protein inhibiting virus release and has antiviral activity. HIV-1 Vpu enhances the release of viral particles from infected cells by counteracting human tetherin [162]. The ExxxLV motif in the second α-helix has been shown to be required for tetherin degradation and virion release from CD4+ T cells [145]. Mutation of the motif (which is conserved in most HIV-1 clades) inhibits the ESCRT-dependent degradation of Vpu-tetherin complex [145]. This transmembrane interaction is required for Vpu interactions with APs [163]. Two other domains in Vpu (Yxxφ and DSGxxS) could mediate anti-tetherin activity [112], whereas the [GD]DIWK motif in monkey BST2, but not in human, is required for interaction with HIV-1 Vpu [113].

### 4.5. Transmembrane Domain (TMD) Interactions

Viral proteins can interact with cellular proteins through TMDs to counteract innate immune response. These interactions are mediated by motifs. HIV-1 Vpu can antagonize tetherin within the lipid bilayer, with α-helical TMDs of both proteins [114]. The conservation of the Ax_3_Ax_3_Ax_3_W and Vx_3_IxxLx_3_L motifs in HIV Vpu and primate BST2, respectively, suggests their putative role in TMD interaction [114,115]. Also, the GxxxG motif is identified for protein-protein, transmembrane-helix and helix-helix interactions [164,165]. Mutation in the ^125^GxxxG^129^ motif in the second transmembrane segments of the NS4B protein may influence protein-folding and interactions, and the replication of engineered HCV-JFH1 [166]. Another example is the influenza virus M2 ion channel protein, which is vital for replication and proton transport [167,168]. M2 has a transmembrane domain, which harbors the conserved HxxxW motif, where H and W are involved in the protein’s channel function. Similarly, the p7 protein encoded by HCV is a viroporin that harbors the HxxxW conserved motif and can transport protons [132].

### 4.6. Retinoblastoma (Rb or pRb)

The Rb encoded by humans is involved in protein-protein interactions, gene expression, cell division and acts as a tumor suppressor. Interaction between oncogenic protein and Rb leads to the phosphorylation and inactivation of Rb, and the progression of cancer. Viral oncoproteins can utilize the conserved Rb-binding motif (LxCxE) on viral proteins to bind to Rb, modulate gene expression, and cause tumor growth. Examples of Rb-binding proteins are as the following: (i) human CMV UL97 serine-threonine kinase [101]; (ii) Polyomaviruses large and small T antigen oncoproteins, which interact with tumor suppressor proteins, and Merkel cell polyomavirus (MCPyV) large T antigen, which harbors LxCxE and NLS (RKRK) motifs (essential for replication) [102,103,104,105,106]; (iii) White spot syndrome virus IE1 and WSV056 that regulate cell cycle progression [107]; (iv) Adenovirus E1A [108]; and (v) HPV E7 [109]. Furthermore, Rb-related protein (RBR) in plants is involved in protein-protein interactions and gene expression [169]. The geminiviruses replication factor AL1 interacts with RBR to modulate host gene expression and DNA replication machinery. It is noteworthy that the LxCxE motif is not the exclusive Rb-binding motif, for instance AL1 does not harbor the LxCxE motif, but recruits helix 4 to bind to plant RBR [169].

### 4.7. Cleavage Site Motif

The viral protein precursor is cleaved by cellular proteases (e.g., matriptase or furin) into active protein form. Among the examples, NDV fusion glycoprotein (F protein) is encoded as an inactive precursor, which is cleaved proteolytically, into two bisulfide-linked polypeptides [170,110]. This cleavage determines the strain type, either lentogenic (avirulent), mesogenic (intermediate) or velogenic (virulent). The consensus sequence of the F protein cleavage site of lentogenic is ^112^[GE][KR]Q[GE]Rα↓L^117^, while the site of velogenic and mesogenic strains is ^112^[RK]RQ[RK]R↓F^117^ [110]. Moreover, the F protein mediates virus entry and fusion with the cell membrane for most avian paramyxoviruses type 9 (APMV-9) strains. Recent reports show that the F protein cleavage site sequence is not a major determinant of pathogenicity and virulence of APMV-7 in chickens [171], and other regions of the F protein could modulate virus virulence [172]. In influenza A virus, the cleavage site of HA is Rx[RK]R↓GLF in highly pathogenic avian influenza virus H5N1, while RxxR↓, RxRR↓, and KKKR↓ are also reported [111]. The R and K can be replaced by non-basic residues, such as [QE][ST]R↓GLF.

## 5. Motifs Essential for Virion Structure and Life Cycle (Usually Unique to Virus Families)

### 5.1. Motifs Involved in Structural Proteins

Adenoviruses bear short and/or long fibers. The fiber consists of a shaft and knob. Analysis of Adv fibers showed that the Adv-D fiber shaft bears fiber flexibility motifs KLGxGLxF[DN] and KxGGLxF[DN], which may have roles in interactions with host cells [50].

### 5.2. Transposition

Kaposin is an oncoprotein that transforms cells in culture and induces tumor formation. Expression and transforming activity of Kaposin A protein is determined by the LxxLL motif [116,117], whereas LQQLL in HIV-1 viral protein of regulation (Vpr) is required for retrotransposition [118,119]. Also, LxxLL and PDZ protein-binding domains are important for the HPV16 E6 protein to interact with the p53 protein [120,173,174,175]. The interaction then activates mTORC1 (rapamycin complex 1) signaling, kinase phosphorylation, translation initiation factor and cap-dependent translation. Therefore, HPV16 E6 protein is correlated with HPV-induced oncogenesis and could be considered as a future therapeutic against HPV-induced cancers [120,121]. Further evidence shows that E6 proteins lacking the LxxLL motif can interact with p53 [122].

### 5.3. Inhibitor of Apoptosis (IAP) Family Proteins (Apoptosis Suppressors)

**Figure 4:**
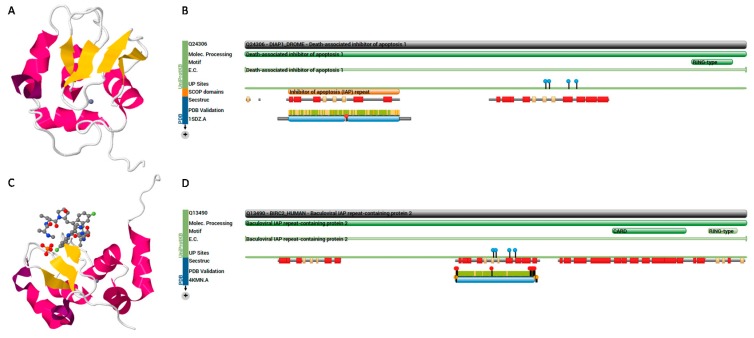
(**A**) Structure of death-associated inhibitor of apoptosis 1 (DIAP1) protein of *Drosophila melanogaster* (PDB ID: 1SDZ, Uniprot ID: Q24306) [179]; (**B**) protein features show that it belongs to the IAP family, and contains two BIR repeats and a RING-type zinc finger; (**C**) structure of baculoviral IAP repeat-containing protein 2 (BIRC2) of human (PDB ID: 4KMN, Uniprot ID: Q13490); (**D**) protein features show that it contains three BIR repeats, a CARD domain and a RING-type zinc finger. The figures adopted from PDB and Uniprot.

IAP is encoded by virus members of eight families: *Ascoviridae*, *Asfarviridae*, *Baculoviridae*, *Hytrosaviridae*, *Iridoviridae*, *Malacoherpesviridae*, *Nudiviridae*, and subfamily *Entomopoxvirinae* of family *Poxviridae* [176]. Baculoviruses block apoptosis by encoding IAPs, which are characterized by the presence of one or more baculoviral IAP repeat (BIR) domains, except for *Deltabaculovirus* [177,178]. The core component of BIR is a Cys/His motif (Gx_2_Yx_4_Dx_3_Cx_2_Cx_6_Wx_9_Hx_6–10_C) that coordinates a single zinc ion; however, about two-thirds of the human IAP proteins harbor a C-terminus RING domain (40–60 amino acids), with consensus Cx_2_Cx_9–39_Cx_1–3_Hx_2–3_Cx_2_Cx_4–48_Cx_2_C [123]. IAP (70 amino acids) mediates protein-protein interactions essential for anti-apoptotic potential [124] by binding to the IAP-binding motif (A[KITV][AEP][FEISY]) (Figure 4, Table S1) [125].

## 6. Motifs Enriched with Residues (Xaa-Rich Motifs) and Low-Complexity Regions

Low-complexity regions (LCRs) are repeats or extensions of one or more residue(s), which could be flanked or interrupted by other residues [180,181,182,183]. Few structural and functional data are available on LCRs, because they may not crystallize easily [181,182,183]. However, they may play roles in protein-protein interactions [183]. In bibliography, there is another type of sequences, which are not referred to as LCRs. They are referred to as Xaa-rich or X-rich motifs, where “X” or “Xaa” refers to any amino acid. They are enriched with residue(s), which may not be repeated, but are flanked by other residues. These alternative residues enrich the structure of x-rich motifs. G-rich residues could be considered as an example, such as GxxxG, [VI]xGxGxxG or (Gx_1–3_Gx_1–3_G). They can be detected in oxidoreductases and may mediate binding to FAD or NAD [128]. Also, the KR-rich motif (such as RKRK and RRRRx_5_Rx_4_RK) is an example which may represent a classic NLS [100]. The functions and structures of these sequences deserve to be elucidated by future studies.

### 6.1. Cys-Rich Motifs

Thioredoxins (trx) belong to the oxidoreductase superfamily, and harbor thioredoxin fold, which is a four-stranded β-sheet surrounded by three α-helices. It reduces thiol groups during thiol-disulfide exchange [184,185,186]. The trx fold first was discovered in bacteria, then found in eukaryotes. The family harbors a conserved CxxC active site motif, which is a signature for the family and thiol-disulfide reactions. CxxC and CxxxC motifs have roles in poxvirus A16 protein interaction and thiol-disulfide transfer during cytoplasmic redox pathway [129]. Moreover, the CxxC motif in the HTLV-1 envelope-fusion protein (env) mediates disulfide isomerization and, hence, promotes viral fusion and infection [130]. CxxxC in Respiratory syncytial virus G protein contributes to virus pathogenicity by binding to the CX3CR1 receptor on host cells [131]. Blocking CX3CR1 with antibodies reduces infection and triggers the immune response.

Proteins containing the chitin-binding domain, or the 6-cysteine motif, Cx_13–20_Cx_5–6_Cx_9–19_Cx_10–14_Cx_4–14_C, are able to degrade chitin and chitotriose. Other proteins have antimicrobial activity and are associated with immune response against pathogens. Ac83 and ha83 proteins encoded by baculoviruses harbor putative C2HC zinc finger (Cx_5_Cx_n_Hx_6_C) and 6-cysteine motifs, respectively, and have a role in budded virion production and nucleocapsid assembly [95,96]. A zinc finger domain is also characterized in the large T antigen of polyomaviruses, including SV40 [106,187]. Large T antigen (LTag) contains four conserved domains, the J domain, the origin-binding domain (OBD), the zinc-binding domain, and the AAA+ ATPase domains. The J domain may have a role in viral DNA replication, OBD may contribute to DNA replication and binding to transcription factors, and ATPase has enzymatic activities to support the required energy, while the zinc finger domain is responsible for the oligomerization of LTag forming hexamers [106,187].

### 6.2. SR-Rich Motif

These LCR motifs are found in a number of viral proteins, which suggests their role in virus replication [188]. Among these proteins are: (1) SSRSSSRSRGNSR in SARS-CoV nucleocapsid protein; (2) RSNSRSRSRSRSRSR and SRSKSRARSQSR in turkey and human astrovirus capsid protein, respectively; (3) SSRYSSTSRERSRLSR in Marburg virus L protein; and (4) RSISRDKTTTDYRSSRS in the minor nucleoprotein of Ebola virus.

### 6.3. PEST Motif

This is a peptide sequence which is rich in Pro (P), Glu (E), Ser (S) and Thr (T). It acts as a signal peptide for protein degradation. The motif is required for binding between the HPV16 E7 protein with human interferon regulatory factor-9 [189]. The PEST motif was predicted in HBV proteins and mouse norovirus non-structural protein; however, the exact role in infection is unknown and may not be necessary for the infection process [190,191].

## 7. Concluding Remarks and Future Perspective

This article reviews the functional motifs utilized by viruses. These motifs are required for productive virus infection. The patterns and functions of motifs were highlighted, aiming to present an insight into motifs and their patterns. The proteins harboring these motifs, as well as viruses encoding these proteins, were also highlighted. The motifs were divided into five main groups according to their cellular function during the virus replication cycle (Figure 1, and as summarized in Table 1).

It worth emphasizing that viruses may use multiple motifs for one process. They might be able to evolve mechanisms to utilize alternative motifs in the absence of the primary one. For example, (i) SUMO-binding to substrate [12,13]; (ii) RGD-like motifs (RGG or GGG) [55]; and (iii) the LxCxE motif is not the exclusive Rb-binding motifs [169]. Moreover, the consensus pattern is not the absolute measure for the protein functions. Although the motif might fulfill the pattern consensus, it could not perform the function. Other factors could influence the function. For example, the NTCP harbors two LL motifs, (^136^LL^137^) and (^222^LL^223^), but the second motif was shown to be more effective in regulating endocytosis [61], which could be due to the phosphorylation of the adjacent T^225^ and S^226^ residues. The ^125^GxxxG^129^ motif in the second transmembrane segments of the NS4B protein, but not ^143^GxxxG^147^ in the third segments, is required for HCV replication [166].

These motifs mediate interactions and molecular processes within host cells. Therefore, an increasing amount of evidence suggests that motifs can be considered as potential targets for therapeutic agents. These attempts include (i) interfering with post-translational modification processes by SENPs proteases [17,18,21]; (ii) motifs mediating the ESCRT pathway (P[TS]AP, PPxY and KATN) as anti-filovirus therapeutic agents [78,81,84,85]; (iii) inhibiting Vif-mediated degradation of antiretroviral A3 [147,133]; (iv) HPV16 E6 protein acting against HPV-induced oncogenesis [120,121]. Moreover, targeting and counteracting proteins (motifs) involved in entry could lead to an efficient therapeutic strategy [192], whereas targeting cellular processes may lead to increased cytotoxicity.

It is also important to emphasize that studying functional motifs would benefit from the prediction of protein characteristics, cellular interactions or the putative role of a protein. The link between functional motifs and protein functional analysis and/or prediction should be established by future research. Moreover, these studies may assist in characterizing virus tropism and studying emerging viruses (zoonotic viruses) capable of infecting humans [56,193]. Since these motifs are subjected to evolutionary modifications, it is of interest to study lateral gene transfer between species or strains as well as evolutionary events occurring in proteins. Also, it is important to study functional and molecular modifications accompanying insertion into or mutation of the motifs within proteins. On the other hand, the numbers of newly isolated viruses were expanded over last years, particularly giant viruses, which harbor proteins of unknown functions. This expansion requires efforts by future research to predict protein functions, which could be achieved by *in silico* determination of sequence characteristics and prediction of structural and functional sites in the sequences prior to designing further experiments.

## Supplementary Materials

The following is available online at www.mdpi.com/2227-7382/4/1/3/s1. Table S1: IAP and nuclear trafficking motifs.
