# Supplementary material for: A Review of Functional Motifs Utilized by Viruses
Source: Proteomes. 2016 Jan 21;4(1):3. doi: 10.3390/proteomes4010003 (PMC5217368; doi:10.3390/proteomes4010003)
Supplement: Supplementary file 1 [file proteomes-04-00003-s001.pdf]

proteomes

Supplementary Martials

A Review of Functional Motifs Utilized by Viruses

Haitham Sobhy

Table S1. IAP and nuclear trafficking motifs.

Description	Consensus Motif	Reference
**IAP Family Proteins-Suppressors of Apoptosis**		
BH1, PS01080; Apoptosis regulator, Bcl-2 family BH1 motif signature (PATTERN)	[LVMENQ][FTLS]x[GSDECQ][GLPCKH]x{1,2}[NST][YW]G[RK][LIV][LIVC][GAT][LIVMF]{2}xF[GSAEC][GSARY]	PROSITE: PS01080
BH2, PS01258; Apoptosis regulator, Bcl-2 family BH2 motif signature (PATTERN)	W[LIM]x{3}[GR]G[WQ][DENSAV]x[FLGA][LIVFTC]	PROSITE: PS01258
BH3, PS01259; Apoptosis regulator, Bcl-2 family BH3 motif signature (PATTERN)	[LIVAT]x{3}L[KARQ]x[IVAL]GD[DESG][LIMFV][DENSHQ][LVSHRQ][NSR]	PROSITE: PS01259
BH4_1, PS01260; Apoptosis regulator, Bcl-2 family BH4 motif signature (PATTERN)	[DS][NT]R[AE][LI]Vx[KD][FY][LIV][GHS]YKL[SR]Q[RK]G[HY]x[CW]	PROSITE: PS01260
LIG_BIR_II_1	^M{0,1}[AS]	ELME000285, http://elm.eu.org/elms/elmPages/LIG_BIR_II_1.html
LIG_BIR_III_1	^M{0,1}AxPx	ELME000279, http://elm.eu.org/elms/elmPages/LIG_BIR_III_1.html
LIG_BIR_III_2	DAxPx	ELME000287, http://elm.eu.org/elms/elmPages/LIG_BIR_III_2.html
LIG_BIR_III_3	^M{0,1}Ax[AP]x	ELME000288, http://elm.eu.org/elms/elmPages/LIG_BIR_III_3.html
LIG_BIR_III_4	DAxGx	ELME000293, http://elm.eu.org/elms/elmPages/LIG_BIR_III_4.html
**Nuclear Trafficking**		
**Nuclear Localization Sequence**		
TRG_NLS_Bipartite_1	[KR][KR].{7-15}[^DE]((K[RK])|(RK))(([^DE][KR])|([KR][^DE]))[^DE]	ELME000276, http://elm.eu.org/elms/elmPages/TRG_NLS_Bipartite_1.html
TRG_NLS_MonoCore_2	[^DE]((K[RK])|(RK))[KRP][KR][^DE]	ELME000270, http://elm.eu.org/elms/elmPages/TRG_NLS_MonoCore_2.html
TRG_NLS_MonoExtC_3	[^DE]((K[RK])|(RK))(([^DE][KR])|([KR][^DE]))(([PKR])|([^DE][DE]))	ELME000278, http://elm.eu.org/elms/elmPages/TRG_NLS_MonoExtC_3.html
**Nuclear Export Signal**		
TRG_NES_CRM1_1	([DEQ].{0,1}[LIM].{2,3}[LIVMF][^P]{2,3}[LMVF].[LMIV].{0,3}[DE])|([DE].{0,1}[LIM].{2,3}[LIVMF][^P]{2,3}[LMVF].[LMIV].{0,3}[DEQ])	ELME000193, http://elm.eu.org/elms/elmPages/TRG_NES_CRM1_1.html
